# Patterning of palatal rugae through sequential addition reveals an anterior/posterior boundary in palatal development

**DOI:** 10.1186/1471-213X-8-116

**Published:** 2008-12-16

**Authors:** Sophie Pantalacci, Jan Prochazka, Arnaud Martin, Michaela Rothova, Anne Lambert, Laure Bernard, Cyril Charles, Laurent Viriot, Renata Peterkova, Vincent Laudet

**Affiliations:** 1 "Molecular Zoology", Institut de Génomique Fonctionnelle de Lyon ; Université de Lyon ; Université Lyon 1 ; CNRS ; INRA ; Ecole Normale Supérieure de Lyon, 46 allée d'Italie, 69364 Lyon Cedex 07, France; 2 Department of Teratology, Institute of Experimental Medicine – Academy of Sciences CR, vvi, Videnska 1083, 14220 Prague 4, Czech Republic; 3 iPHEP – CNRS UMR 604, UFR SFA, Université de Poitiers, 40 avenue du recteur Pineau, 86022 Poitiers Cedex, France; 4 "Evo-Devo of Vertebrate Dentition", Institut de Génomique Fonctionnelle de Lyon ; Université de Lyon ; Université Lyon 1 ; CNRS ; INRA ; Ecole Normale Supérieure de Lyon, 46 allée d'Italie, 69364 Lyon Cedex 07, France

## Abstract

**Background:**

The development of the secondary palate has been a main topic in craniofacial research, as its failure results in cleft palate, one of the most common birth defects in human. Nevertheless, palatal rugae (or *rugae palatinae*), which are transversal ridges developing on the secondary palate, received little attention. However, rugae could be useful as landmarks to monitor anterior/posterior (A/P) palatal growth, and they provide a simple model of mesenchymal-epithelial structures arranged in a serial pattern.

**Results:**

We first determined in which order the nine mouse rugae appear during development. Our results revealed a reiterative process, which is coupled with A/P growth of palatal shelves, and by which rugae 3 to 7b are sequentially interposed, in the increasing distance between the second most anterior ruga, ruga 2, and the two most posterior rugae, rugae 8 and 9. We characterized the steps of ruga interposition in detail, showing that a new ruga forms from an active zone of high proliferation rate, next to the last formed ruga. Then, by analyzing the polymorphism of wild type and Eda^Ta^ mutant mice, we suggest that activation-inhibition mechanisms may be involved in positioning new rugae, like for other skin appendages. Finally, we show that the ruga in front of which new rugae form, i.e. ruga 8 in mouse, coincides with an A/P gene expression boundary in the palatal shelves (*Shox2*/*Meox2-Tbx22*). This coincidence is significant, since we also found it in hamster, despite differences in the adult ruga pattern of these two species.

**Conclusion:**

We showed that palatal rugae are sequentially added to the growing palate, in an interposition process that appears to be dependent on activation-inhibition mechanisms and reveals a new developmental boundary in the growing palate. Further studies on rugae may help to shed light on both the development and evolution of structures arranged in regular patterns. Moreover, rugae will undoubtedly be powerful tools to further study the anteroposterior regionalization of the growing palate.

## Background

**Figure 1:**
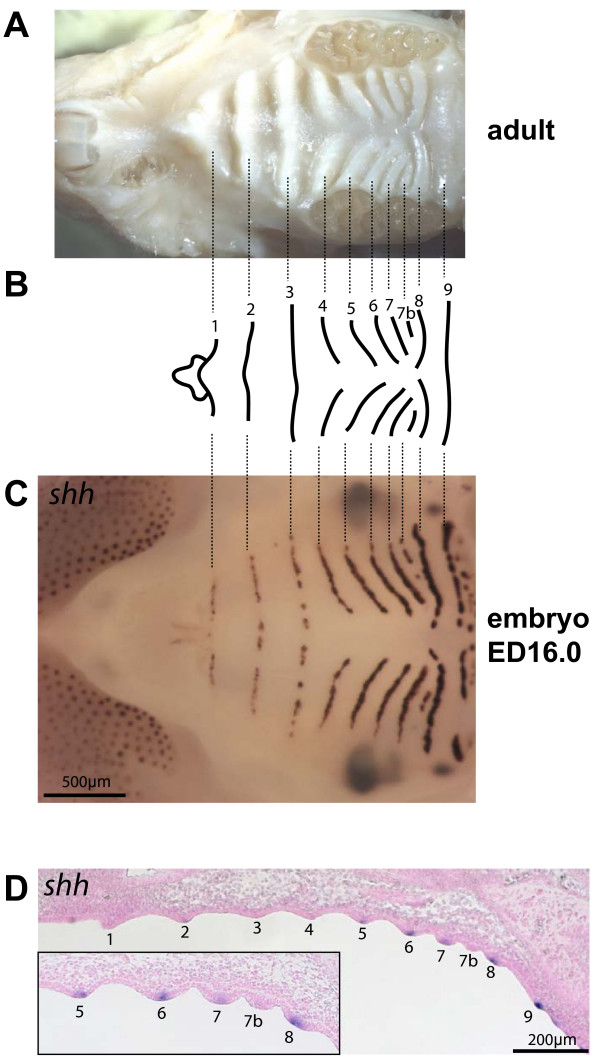
**Mouse adult ruga pattern and its visualization in the fetus by *in situ *hybridization against *Shh *gene**. (A) The roof of the oral cavity of an adult mouse showing the palatal ridges (*rugae palatinae*) on the hard palate. (B) Mouse rugae pattern with numbering used in this study. Note that ruga 7b was called 8b in other studies (Peterkova et al. 1987; Charles et al. 2007). (C) In ED16.0 fetus, *Shh *gene expression pattern (as seen by whole-mount *in situ *hybridization) prefigures the adult ruga pattern. (D) Sagittal section through the same embryo as in C, showing *Shh *expression in the epithelium at the tip of rugae (see magnification in the low left corner). The absence of *Shh *signal in the rugae 1–4 can be explained by its discontinuity in the anterior rugae at this stage (see C).

The development of the mammalian secondary palate is a critical process whereby two bilateral outgrowths of the embryonic maxilla (the palatal shelves) come to fuse at the midline to separate the nasal from the oral cavities. Failure of this process is responsible for cleft palate, one of the most common birth defects in human. That is why the development of the mammalian secondary palate has been extensively studied in the last past thirty years (reviewed in [1]). Secondary palate can be divided in two parts, depending on the nature of the underlying structure: the hard palate, which is ossified (with contribution from two bones: maxilla and palatine), and the posterior soft palate, which is muscular. Both hard and soft palates are covered with a squamous pluristratified epithelium on their oral side. Palatal ridges or *rugae palatinae *are transversal ridges found on the hard palate of most mammalian species, but their number and arrangement are species specific [2]. Laboratory mouse strains have at least nine rugae, a tenth ruga (ruga 7b, Fig. 1) being more or less frequently present, depending on the strain [3,4]. Together with the teeth and the tongue, rugae take part in mastication by helping to sense, hold and mash the food (for review see [4]). Indeed, rugae harbor various types of intraepithelial sensory structures (such as Merckel cells, corpuscular endings and free nerve endings, [5]) and play a sensory role when the food is pressed by the tongue against the hard palate [6]. Moreover, in animals where they are very prominent (e.g. ruminants), rugae also have a mechanical function by helping in mastication and preventing slicing of a mouthful [7].

Compared with secondary palate development, which has been extensively studied, rugae development received little attention. Using electron microscopy scanning and histological sections, Peterkova et al. [4] followed rugae development in mouse soon after palatal shelves begin to form (Embryonic Day = 12.5) to the end of prenatal development and defined 5 stages: I – thickened epithelium burrowed into mesenchyme; II – protruding epithelium with condensed mesenchyme; III – epithelial and condensed mesenchymal cells specifically arranged in a manner resembling magnetic lines of power; IV – transition from primitive to definitive ruga: the fibrous stroma has formed and vaults in the oral direction the epithelium that becomes thinner; V – specific orientation of cells remains visible, the epithelium has started to keratinize and shows similar thickness to the adjacent oral epithelium. Ruga morphogenesis may be less spectacular than that of classically studied skin appendages (such as tooth, hair and feather). However, it shares obvious similarities in very early development with all skin appendages, and as development proceeds further, with a subset of them. Namely, the earliest steps of ruga development (thickening of the epithelium and condensation of the underlying mesenchyme) are similar in all skin appendages. However, the ruga primordium, a protruding epithelium associated with a condensed mesenchyme, more closely resembles a feather or scale primordium, than a hair or tooth primordium (because in this case the epithelium is invaginated). Later on, the epithelium is vaulted and, on sagittal section, rugae are then similar to scales. Since hair, tooth but also feather and scale early development involves some common pathways [8,9], one can expect that rugae will be no exception. Indeed, there are some indications that key players of skin appendage development may be involved in rugae development: rugae pattern is disrupted in *EctodysplasinA (Eda) *mutant mice [3] and rugae are lost in *Fgf10 *mutant mice [10].

Peterkova et al. [4] have shown that mouse anterior rugae develop first, but could not establish the precise ranking of rugae appearance by using only morphological criteria. Knowing this ranking would however allow the use of rugae as anterior/posterior (A/P) landmarks in the growing palate. While A/P regionalization and growth of the palate had been neglected in former studies, these aspects have recently received more attention, as underlined in recently published reviews [1,11]. The best described example is the anterior *Shox2 *gene and the posterior *Meox2 *gene, which are mutually exclusive [12-14]. Nevertheless data are still scarce, notably due to the difficulties in comparing gene expression borders from one study to another, or even within the same study, since mouse embryos of similar age can show significant differences in developmental stage (for review see [15]). The study of rugae development could help to rationalize these data and contribute to giving palate development a third (A/P) dimension. In the same way, this may also be beneficial to developmental studies devoted to teeth that are close neighbors of rugae.

Apart from their interest as morphological landmarks, rugae are also interesting for three additional reasons. First, study of their development may bring new insights into palatogenesis. Indeed, embryonic rugae have long been suspected to help palatal shelve elevation, the process whereby palatal shelves, which had been growing down the sides of the tongue, suddenly elevate to a horizontal position above the dorsum of the tongue in order to subsequently fuse along the midline. Several authors proposed that embryonic rugae could stiffen palatal shelves and even that cell reorganization in rugae might provide part of the forces needed to horizontalize palatal shelves [16-18]. Second, rugae provide a new and simple model for studying skin appendage formation, as compared with tooth, hair and feather that have long been studied but undergo a much more complex morphogenesis. From an evo-devo perspective, adding a new model to this list would further illuminate how similar, yet different, genetic networks are used in skin appendage development to result in different shapes [8,19]. Finally, when considering their regular pattern, rugae provide a new model to study how regular arrangement of serial organs is achieved during development.

For all these reasons, we decided to investigate rugae development in more depth. First, we used *Shh *as a molecular marker to establish a precise temporal sequence for rugae appearance in mouse. Our results revealed a reiterative process, by which new rugae are sequentially interposed between the last formed ruga and the second last posterior ruga in the adult mouse, ruga 8. We characterized this interposition process in detail and we provide data suggesting that activation-inhibition mechanisms are involved. Finally, using both mouse and hamster, we show that this process reveals a conserved A/P boundary for palatal development.

## Results

### Shh expression reveals a sequential order for ruga appearance

**Figure 2:**
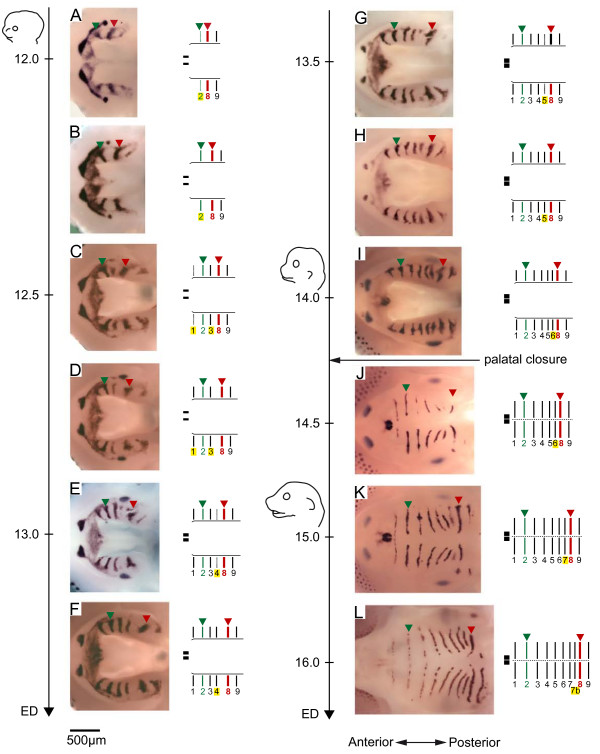
**Sequence of mouse ruga appearance as determined by *Shh *expression pattern**. Embryos were ranked according to their weight (A: 80–90 mg; B-: 95 mg; C: 120–130 mg; D: 120–130 mg; E: 130–140 mg; F: 160–170 mg; G: 160–170 mg; H: 190–200 mg; I: 270–280 mg; J: 300–310 mg; K: 370–380 mg; L: 590–600 mg). Age of embryos (ED = embryonic day) is shown along the time scale. Dissected upper jaws hybridized with a mouse *Shh *probe are shown (left), together with a corresponding scheme showing ruga numbering (right). In this scheme, starting *Shh *expression is represented with dotted lines and the new forming (latest) ruga is underlined in yellow. Drawings of mouse heads are shown at ED 12.0, 14.0 and 15.0 to underline that the A/P elongation of the region delimited by ruga 2 (in green) and ruga 8 (in red) parallels the elongation of the snout. Note that in some cases ruga 9 has been removed during separation of the upper from the lower jaw.

In late embryos (ED16.0), the *Shh *gene was expressed in rugae and its expression pattern closely mimicked the adult rugae pattern (compare panel C with panel A in Fig. 1). By looking at earlier stages, we found that the striped expression pattern of *Shh *appeared from very early during development (Fig. 2). As seen on sections, the stripes always corresponded to epithelial cells at the tip of rugae, that at least had reached stage I, i.e. thickened epithelium (for examples, see sections later in figure 5). We reasoned that we should be able to determine the order of appearance of *Shh *stripes by simply looking at a series of staged embryos. For this purpose we needed a reliable series of closely consecutive developmental stages, and to achieve this, we used the staging method proposed by Peterka et al. [15], in which embryo weight (in mg) allows a more precise specification of the developmental stage of embryos exhibiting the same chronological age (in ED = Embryonic Day). Then, starting with ED16.0 and looking back in developmental time to the first rugae initiation, we could identify *Shh *stripes according to numbering of rugae in adults. This method allowed us to trace the developmental fate for each ruga. For clarity however, we present here the results according to progressing developmental time (Fig. 2).

From very early stages and then on (ED12.0–16.0), the sharpest stripe of *Shh *expression corresponded to ruga 8 (shown in red, Fig. 2). Ruga 8 seemed to be the first ruga to appear, but it was very rapidly followed by two stripes corresponding to ruga 2 (in green, Fig. 2A) and ruga 9. From then on, new *Shh *stripes emerged between the last formed ruga and ruga 8. For example, ruga 3 emerged between rugae 2 and 8 (Compare Fig. 2B and 2C), ruga 4 between rugae 3 and 8 (Fig. 2D to 2F, note *Shh *expression was just beginning in Fig. 2E), ruga 5 between ruga 4 and 8 (Fig. 2F to 2H, note *Shh *expression was just beginning in Fig. 2G), and so on, until the last ruga was formed, *i.e. *ruga 7b (Fig. 2K to 2L; please note that we called this ruga "7b", instead of "8b" as in previous studies, as it was more consistent with order of appearance [3,4]). The only exception was the stripe corresponding to ruga 1 which emerged anteriorly to ruga 2 and almost contemporary with ruga 3 (Fig. 2C to 2E).

In conclusion, this analysis of *Shh *expression allowed us to define a temporal sequence for ruga appearance that is 8-(2,9)-(1,3)-4-5-6-7-7b. Thus, rugae 3 to 7b are consecutively interposed between the last formed ruga and ruga 8. This process, that we call "interposition process", is accompanied by a clear anteroposterior extension of the region between ruga 2 and 8, by comparison to the region between ruga 8 and 9 (compare Fig. 2A and 2L). Moreover, it closely follows the anteroposterior elongation of the whole jaw (see drawings of embryo heads on Fig. 2). During the rest of our study, we chose to focus on the "interposition process" of rugae 3–7b in front of ruga 8, because of the very interesting reiterative aspect of this process and because of its implications for palatal A/P growth.

### During the interposition process, new rugae are added in the region immediately posterior to the last formed ruga

As the increasing rugae number results from reiterated steps of ruga interposition, we thought that a detailed description of one of these steps should give us a general understanding of the process. We decided to focus on ruga 5 interposition at ED13.5. This allowed investigation of rugae at different stages (ruga 5 being the youngest) and avoiding potential interference with the horizontalization process (which starts at ED14.0).

**Figure 3:**
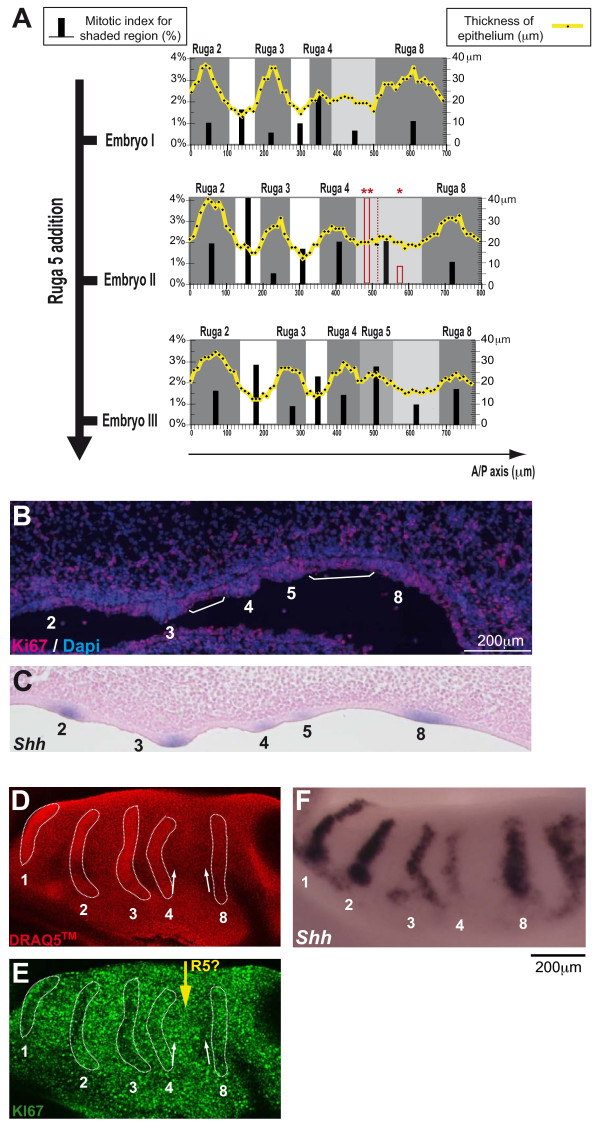
**Proliferation in the palatal shelf epithelium**. (A) For three embryos at three consecutive stages during ruga 5 addition (ED13.5), a graph is given correlating epithelium thickness and mitotic index along the anterior/posterior (A/P) axis of the palatal shelf. The individual measures of epithelium thickness correspond to black points, joined with a thick yellow line. The mitotic index of the epithelium was calculated for different regions as figured in grey (ruga region), white (gap region) or light grey (region of interest where ruga 5 appears). It is indicated with black bars. For embryo II, an extra mitotic index was also calculated for the anterior third (two red stars) and the posterior two-thirds (red star) of the region of interest and is indicated with red bars. (B) Proliferation along the A/P axis of the palatal shelf as visualized on sagittal cryosections with the KI67 antibody at ED13.5 (ruga 5 formed) for an embryo of similar weight as embryo III in A. Ki67 postive cells (pink) are proliferating cells, while Ki67 negative cells (blue, dapi nuclear staining) are quiescent cells. White brackets point the ruga3-ruga4 gap and the region between the last formed ruga (5) and ruga 8. (C) For an equivalent embryonic stage and sectioning plane as in B, sagittal section of an embryo after whole mount *in situ *hybridization with the *Shh *probe. (D) A dissected palatal shelf at ED13.5 (ruga 5 in formation) was stained with the nuclear marker Draq5TM. The cells are more densely packed in well-formed rugae, allowing their visualization. (E) The same palatal shelf was immunolabeled with Ki67 to show proliferation in the epithelium. Note that Ki67 negative cells are found mainly in rugae (especially in more mature rugae 1–3) and in the region immediately anterior to ruga 8 (compare labeling in the regions pointed by the two white arrows). (F) A littermate embryo of similar weight was hybridized with the *Shh *probe to help staging. The large space between ruga 4 and 8 confirmed that ruga 5 is already developing, as for embryo II in A.

We first chose three embryos at three close developmental stages, which corresponded to ruga 5 interposition (ED13.5; embryos I, II, III in Fig. 3A). By counting mitotic and non-mitotic epithelial cells and measuring epithelium thickness on every two second frontal sections of the palatal shelf, we could determine the mitotic index (i.e. a number of mitotic cells per 100 cells) along the A/P axis of the palatal shelf, that is, in gap areas (white in Fig. 3A), in rugae areas (dark grey) and in the region of interest, anterior to ruga 8 (light grey). We found a higher mitotic index in the gap than in the rugae areas (see the gap between ruga 2 and ruga 3 or between ruga 3 and ruga 4, Fig. 3A). Moreover, the mitotic index of ruga 4 progressively decreased from embryo I, where ruga 4 was only poorly developed, to embryo III, where it was fully developed, suggesting that proliferation is progressively decreasing as ruga outgrowth is completed.

In the region anterior to ruga 8 (light gray), the epithelium was only a little bit thicker than in gap areas, and curiously, the mitotic index was low, except for embryo II. In this case however, we noticed that the repartition of mitosis was strongly biased in favor of the anterior third of the region (see the mitotic index in red). This might correspond to the formation of ruga 5, since in the older embryo III, ruga 5 thickening was now discernable just next to ruga 4. In conclusion, ruga 5 seems to form from a burst of proliferation in the region immediately posterior to ruga 4, while the region immediately anterior to ruga 8 tends to proliferate at lower level. A true gap between ruga 4 and ruga 5 is only formed later on (see later at ED14.0 on figure 5).

In order to confirm these results, we used the Ki67 antibody to label head sagittal cryosections of embryos showing similar weight to the embryo III (Fig. 3B–C). This antibody is known to label cells engaged in the cell cycle, but not quiescent cells. Moreover it discriminates interphasic cells (discrete nucleolar signal) from mitotic cells (very sharp signal), and can thus be used as an indicator of proliferation activity. Results were consistent with the quantitative evaluation of mitotic index. Quiescent cells (non labeled cells) were found mostly in rugae, thus explaining their overall low mitotic index (Fig. 3B). The forming ruga 5 had more mitotic cells as compared with other rugae, in agreement with its higher mitotic index. Finally, it seems that the gap region just in front of ruga 8 had more quiescent cells than other gap regions (compare with the gap between ruga 3 and 4, white brackets), in agreement with a low mitotic index.

To complete these results obtained on sagittal sections, we used a complementary, whole mount approach. We performed whole-mount immunochemistry with Ki67 antibody and visualized the surface epithelium with confocal microscopy (Fig. 3D–F). Post-staining with DRAQ5TM allowed visualization of cell nuclei. Therefore the rugae could be well detected, possibly because cells are more densely packed there (Fig. 3D). This pattern closely resembled that seen through *Shh *expression in a littermate embryo of similar weight (Fig. 3F). As judged from the distance between ruga 4 and ruga 8, the stage seemed equivalent to that of "embryo II" in Fig. 3A. Results were again fully consistent with counting of mitotic index: unlabeled, quiescent, cells were mostly found in rugae and in the region next to ruga 8; on the other hand labeled, proliferating, cells were mostly found in gaps and in the region next to ruga 4, where ruga 5 will form (compare the two white arrows in Fig. 3E). We concluded that epithelial proliferation is high in all gaps, except in a quiescent zone just anterior to ruga 8. In contrast, proliferation is low in rugae, except in the new ruga, which seems to form from an active zone just posterior to lastly formed ruga.

### Observations on wild type and Eda^Ta ^mutant strains suggest that formation of a new ruga is only allowed at a distance from flanking rugae

In a previous study, we studied variations of the ruga pattern in Eda^Ta ^mice and in their wild type counterpart [3]. These variations had drawn our attention because they revealed morphological correlations between consecutive rugae.

**Figure 4:**
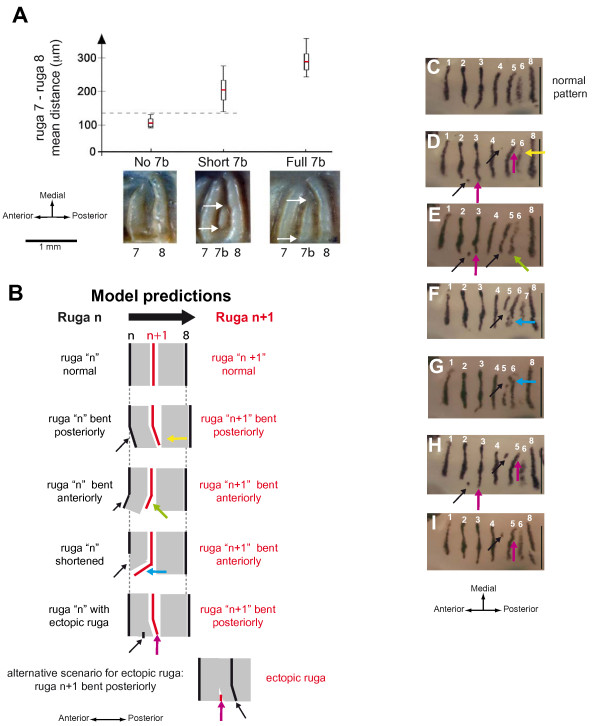
**Interposition of a new ruga depends on the distance between previously formed rugae**. (A) In wild type mouse adult palates, presence of ruga 7b, and in case of presence, full extension of ruga 7b, are correlated with higher distances between its neighbors, rugae 7 and 8. Pictures show the 3 possible states of ruga 7b: absent (no 7b), short (short 7b) or fully developed (full 7b). Adult half palates of wild type mice were distributed into three classes according to ruga 7b state and the inter-rugae distance between rugae 7 and 8 was measured (see material and methods for details). For each class, the graphics shows the mean inter-rugae distance value (red line), the standard deviation (white rectangle) and the range of variation (vertical black line). (B) In a model where a new ruga can only form at a threshold distance from pre-existing rugae, the shape of "ruga n" allows prediction of that of ruga "n+1". A simplified scheme of ruga "n+1" interposition is shown with ruga n, ruga 8 (in black) and the new ruga "n+1" (in red). In the model, formation of ruga is only allowed at a threshold distance from pre-existing rugae, i.e. out of grayed fields. An abnormal shape of ruga "n" (black arrow) will change the shape of the grayed field, and consequently the shape of ruga "n+1" (color arrow). (C – I) In Eda^Ta ^embryos, propagation of ruga shape anomalies from ruga "n" to ruga "n+1" fit with the previous model. The pictures show half palates of ED14.5–15.0 embryos heterozygous (C, E – I) or homozygous (D) for the Eda^Ta ^mutation and hybridized with *Shh *probe. As in B, paired arrows point the abnormal shape of ruga "n" (black arrow) and the associated abnormal shape of ruga "n+1" (color arrow, color according to B). For each picture, scale bar is 500 μm.

First, in the wild-type sample we studied, ruga7b was highly polymorphic, as reported for other wild type backgrounds [4,20]. It was either absent, short or fully formed (respectively in 9%, 56% and 35% of palatal halves Fig. 4A). As shown above, ruga 7b forms last, between ruga 7 and ruga 8 (Fig. 2). We found that its presence was associated with a higher distance between ruga 7 and 8 (Fig. 4A). A clear threshold in distance was systematically exceeded in half palates with ruga 7b and never reached in half palates without 7b (Fig. 4A). Moreover, whether ruga 7b was fully formed was also clearly correlated with higher distance between ruga 7 and 8 (Fig. 4A). Finally, variations in medio-lateral position and shape of ruga 7b also correlated with variations of the distance between ruga 7 and 8 (arrows in Fig. 4A). In summary, presence of ruga material correlated with exceeding a threshold distance between ruga 7 and 8.

These observations suggest a model where formation of a new ruga "n+1" somehow depends on the distance to its neighboring rugae at the time it forms, i.e. rugae "n" and 8. If true, then we expect that any mis-positioning of ruga "n" should be propagated to ruga "n+1" (Fig. 4B). We had seen this kind of correlations in the Eda^Ta ^mice, in which patterning anomalies are frequently found in adults [3]. These anomalies occur non symmetrically and include ectopic rugae, short rugae and mispositioning disrupting symmetry. From then on, our purpose was not to decipher how these anomalies occur, but whether and in which way an anomaly on ruga "n" is linked with an anomaly of ruga "n+1". According to our putative model, several predictions can be made depending on the type of anomaly in ruga "n" (Fig. 4B), which can be tested in Eda^Ta ^mice. In order to detect even subtle patterning anomalies, we chose to look at the *Shh *pattern in late embryos (ED14.5–15.0) rather than at the adult ruga pattern.

In a model where ruga formation is only allowed at a distance from preexisting rugae (i.e. out of grayed regions in Fig. 4B), an abnormal bending (towards posterior or anterior) of ruga "n" should be propagated to ruga "n+1". For example, ruga "n" bending towards posterior should repel ruga "n+1" formation (both in space and time: until ruga n to ruga 8 distance is wide enough); on the contrary, ruga "n" bending towards anterior should precipitate ruga "n+1" formation. Both cases were found in tabby mice (Fig. 4D–E; yellow and green arrows, compare with the normal pattern in 4C). Then, the model predicts that a shortening of ruga "n" should result in bending of corresponding extremity of ruga "n+1" towards the free place anterior (Fig. 4B). In Eda^Ta ^mice, shortening of ruga 5 was indeed followed by a bending of ruga 6 towards the anterior (Fig. 4F–G; blue arrows). Finally, a small patch of ectopic ruga posterior to ruga "n" should repel ruga "n+1" formation and result in ruga "n+1" posterior bending. Alternatively, such an ectopic patch may form after its flanking rugae, because at this site, the two of them are abnormally distant and consequently fail to prevent ruga formation. Whatever the case, ectopic patches should be found associated with ruga bending (Fig. 4B). In Eda^Ta ^mice, small ectopic patches were eventually found between ruga 2 and 3 (Fig. 4D, 4E, 4H; pink arrows) and ruga 4 and 5 (Fig. 4D, 4H, 4I; pink arrows). In each case, the lateral extremity of ruga 3 and ruga 5 were abnormally bent towards the posterior (compare with Fig. 4C which represents the normal pattern). In conclusion, all these observations are consistent with a model where ruga interposition is only allowed at a certain distance from the flanking rugae.

### Ruga 8 marks a boundary of gene expression in the developing palate

**Figure 5:**
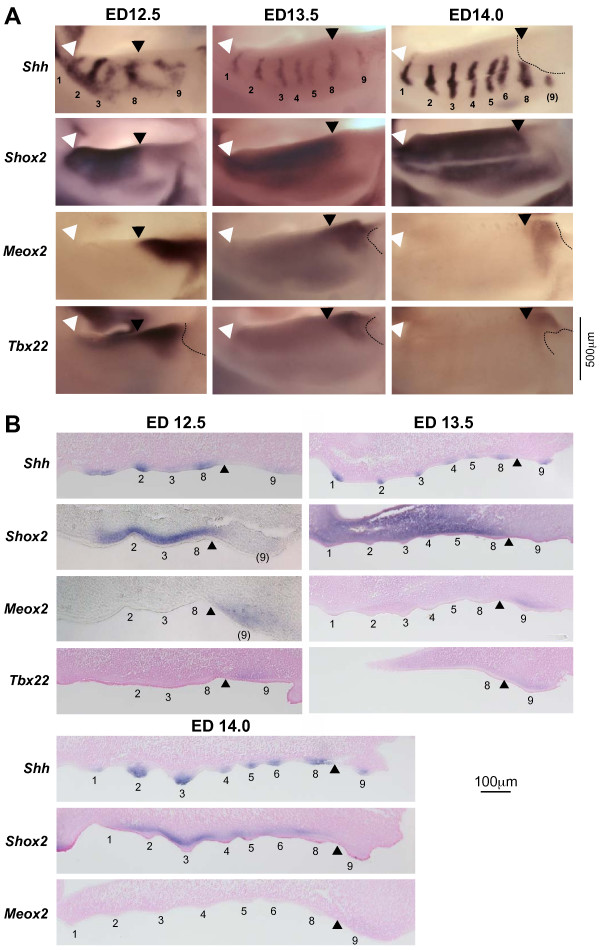
**Anteroposterior limits of expression of *Shox2*, *Meox2 *and *Tbx22 *genes coincide with ruga 8 in the mouse**. Whole mount in situ hybridization for *Shh*, *Shox2*, *Meox2 *and *Tbx22 *were performed on dissected mouse upper jaws at three embryonic stages (ED12.5, ED13.5 and ED14.0, embryos of similar weight classes) (A), and were followed by sectioning, and counterstaining with nuclear red (except for *Shox2 *and *Meox2 *at ED12.5) (B). Rugae are numbered (with brackets when damaged by dissection). The white arrowhead points the anterior limit of the palatal shelf and the black arrowhead points ruga 8. The dashed line indicates where the very posterior part of the shelf was damaged by dissection.

As shown by the previous observations, ruga 8 has a very special position in the palatal shelf and we wondered if it may delimit different territories in the developing palate. We thus looked at expression of three genes, *Shox2*, *Meox2 *and *Tbx22*, that are known to be differentially expressed along the anterior/posterior axis [12,14,21,22]. Interestingly, whatever the embryonic stage we looked at (ED12.5, ED13.5, ED14.0), *Shox2 *was found expressed in the mesenchyme anterior to ruga 8 (including the mesenchyme directly adjacent to ruga 8), while *Meox2 *and *Tbx22 *were always found expressed in the mesenchyme just posterior to ruga 8 (Fig. 5). Thus, the limit of expression of *Shox2*, *Meox2 *and *Tbx22 *genes in the underlying mesenchyme coincides with ruga 8 at consecutive embryonic stages, at least before palatal shelves elevation. Taken together, these data suggested that ruga 8 marks a developmental boundary delimiting two distinct parts of the palatal shelves.

### The golden hamster ruga 7 is equivalent to mouse ruga 8 and marks the same boundary of gene expression

**Figure 6:**
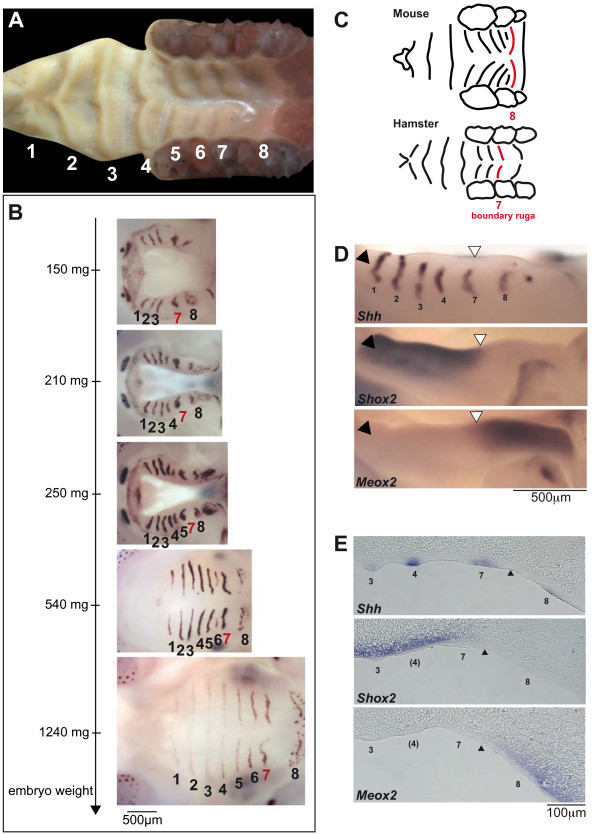
**Ruga 7 in hamster is equivalent to ruga 8 in mouse and also coincides with A/P *Shox2*/*Meox2 *expression boundary**. (A) Ruga pattern and numbering in the hamster *Mesocricetus auratus*. (B) Sequence of hamster ruga appearance as followed with *Ma-Shh *probe. Embryos were ranked according to their weight. (C) Drawings of mouse and hamster palate showing that the boundary ruga is found more anteriorly in the hamster by comparison to the mouse. Drawings were made from pictures shown in Fig. 1A and 6A. Scale was adapted for comparison. (D) Whole mount *in situ *hybridization of hamster embryonic upper jaws (weight class: 170–180 mg) with *Ma-Shh*, *Shox2 *and *Ma-Meox2 *probes. (E) Sections of hamster embryonic upper jaws following whole mount hybridization with *Ma-Shh *(weight class: 170–180 mg), *Shox2 *and *Ma-Meox2 *(weight class: 160–170 mg) probes.

In order to further explore the possibility that mouse ruga 8 indeed represents an important developmental boundary, we looked at rugae development in a distant murid, the golden hamster (*Mesocricetus auratus*). Its posterior palate morphology is quite different from the mouse (Fig. 6A). This species harbors only 7 obvious rugae and a smooth eighth one bordering the soft palate. As for the mouse, we used a probe against the *Shh *gene (ma *Shh*) to determine the order of rugae appearance. Due to difficulties in detecting pregnant females at young embryonic stages, we could not determine the order of appearance for the earliest rugae, and our series started with embryos having already 5 *Shh*-stripes corresponding to rugae 1,2,3,7,8. From then on, rugae were progressively added by interposition between the latter formed ruga and ruga 7 (Fig. 6B), resulting in the partial sequence (1,2,3,7)-4-5-6. Ruga 7 in hamster is thus functionally equivalent to ruga 8 in mouse, and we called these very special rugae the "boundary rugae".

As compared with the mouse, the boundary ruga is found relatively more anteriorly than in the mouse (Fig. 6C). Nevertheless, in hamster as in mouse, the boundary ruga corresponded to the boundary between *Shox2*/*Meox2 *expression in embryos of weight class 190–200 mg (Fig. 6D, and see sections in Fig. 6E). We concluded that, since it is found in two species with different ruga number and pattern, the overlap between the boundary ruga and the *Shox2*/*Meox2 *boundary is unlikely to be fortuitous. Rather, it strongly suggests that the boundary ruga marks a conserved developmental boundary in the murid palate.

## Discussion

### Murid rugae appear through posterior interposition

As many other mesenchymal-epithelial organs, palatal rugae show *Shh *expression at an early stage of their development [23]. However, in rugae, this expression also persists during most stages of their development (Fig 2). In the present paper, we used this early and persistent marker to decipher the sequence of mouse palatal rugae formation from an early stage where only ruga 2, 8 and 9 are clearly discernable. We showed that mouse palatal rugae 3 to 7b form in a sequential manner, whereby they are interposed between the last formed ruga and ruga 8: first ruga 3 between ruga 2 and 8, then ruga 4 between ruga 3 and 8, and so on (Fig. 2). We called this process "posterior interposition", and we called ruga 8 the "boundary ruga". We further showed that the same process is found during the development of another murid rodent, the golden hamster *Mesocricetus auratus*, but in that case less rugae are present and ruga number 7 is the boundary ruga. Variations in number of rugae are common between close rodent species (see the monograph by Eisentraut [2] or variations in species of the *Praomys *complex [24]). They are even common among members of the same species, as exemplified here with ruga 7b of the laboratory mouse, or as also for wild rodent species [2]. These variations could be explained in the light of the interposition process: adding or removing one ruga may be easy to achieve, since it just involves one step more or one step less in the reiterative process of ruga interposition.

### Ruga addition proceeds from an active zone at the posterior end of the last formed ruga

In order to better understand the mechanism of ruga interposition, we focused on close developmental stages around ED 13.5, from morphological appearance of ruga 4 to that of the next ruga, ruga 5.

First, we showed that the proliferation rate progressively decreased in the epithelium of ruga 4 as it became thicker, and finally reached the low level seen in older rugae 2 and 3. By opposition and, as shown previously [25,26], gaps between rugae showed a higher proliferation rate, consistent with the progressive moving away of rugae. The well formed embryonic ruga or "primitive ruga" (as defined by Peterkova in [4]) thus appears as a protruding thickened epithelium comprising cells with a low proliferation level. Of note, *Shh *expression is turned on only lately during formation of this primitive ruga, since it was always found associated with clear thickened epithelium. Interestingly, during tooth development, an epithelial signaling center known as the "enamel knot" is also formed by low (non)-proliferating and densely packed cells expressing *Shh *[27]. Moreover, another characteristic of the enamel knot, namely the presence of apoptotic cells, was also noted (in ruga 2 and 3 at ED13.5, M.R. data not shown – see also [28]). The enamel knot signals to the underlying mesenchyme and to the adjacent growing epithelium [27]. On a sagittal section, the primary ruga with the growing gap epithelium on both sides may thus be compared to the middle ridge of a tooth cap comprising the enamel knot and the adjacent growing epithelium.

Then, we showed that ruga 5 morphologically appears just next to ruga 4, from a region of intermediate epithelium thickness, which underwent a burst of proliferation. In contrast, the region situated immediately anterior to ruga 8 seemed silent: it showed a constant low proliferation rate. Importantly, what we showed here for ruga 5 probably holds true for other interposed rugae, since new rugae are always found closer to their predecessor than to ruga 8 (see with *Shh *stripes on Fig. 2, or on sections Fig. S2). Ruga formation seems thus to proceed by a burst of cell proliferation induced next to the last completed primitive ruga. But what exactly is this burst of proliferation responsible for? Indeed, ruga formation involves two merged events: i) A/P extension of the region between the last formed ruga and ruga 8 (in light grey in Fig. 3A) and ii) the formation of a new ruga (i.e. local thickening of the epithelium) in the free space. In principle, the burst of proliferation should participate at least in thickening since it just precedes it in time (Fig. 3A). It is less clear whether it also participates in A/P extension, since at the time when we observed the mitotic burst, the A/P extension was already obvious (see Fig. 3A embryo 2 or Fig. 3C). However, since cell growth precedes cell mitosis, the A/P extension could in principle be seen before the mitotic burst itself. Alternatively, other mechanisms may also have been involved in this A/P extension, among which for example changes in cell shape or cell rearrangements. Further studies will be necessary to understand the precise dynamics of this region, and how the mechanism of ruga interposition is linked to A/P growth of the palate and of the whole jaw.

### Positioning of a new ruga likely involves activation-inhibition mechanisms

We showed that normal patterning of rugae, and response of the patterning system to anomalies in one ruga, are fully consistent with a model according to which ruga formation is only allowed at a threshold distance from other rugae. Generation of such threshold distances for organ induction often relies on the antagonist activity of activators and inhibitors, in mechanisms known as "activation-inhibition". Of note, such mechanisms have been classically involved in spacing of other ectodermal organs, such as hair and feather [29-31], and they are followed by *Shh *expression in the newly formed placode [8]. Based on our data and by analogy with hair and feathers, we propose that activation-inhibition mechanisms are involved in positioning new rugae during the interposition process, which finally express *Shh*. From then on, we can use our knowledge of these mechanisms in hair and feathers to point candidate pathways to explore in future studies.

Activation of the Wnt/β-catenin pathway is a key early event for early formation and spacing of hair and feathers [9,31,32]. During ruga interposition however, we could not detect any activation of the Wnt/β-catenin pathway as revealed by the TOP-GAL reporter transgene (AM and SP, data not shown), whereas we found activation in hair and tooth, as previously reported [33,34]. Even if we cannot rule out that the TOP-GAL reporter failed to report Wnt/β-cat pathway activation in rugae while it did correctly in neighboring teeth, this preliminary finding suggests that the canonical Wnt/β-cat pathway is not involved in ruga interposition.

**Figure 7:**
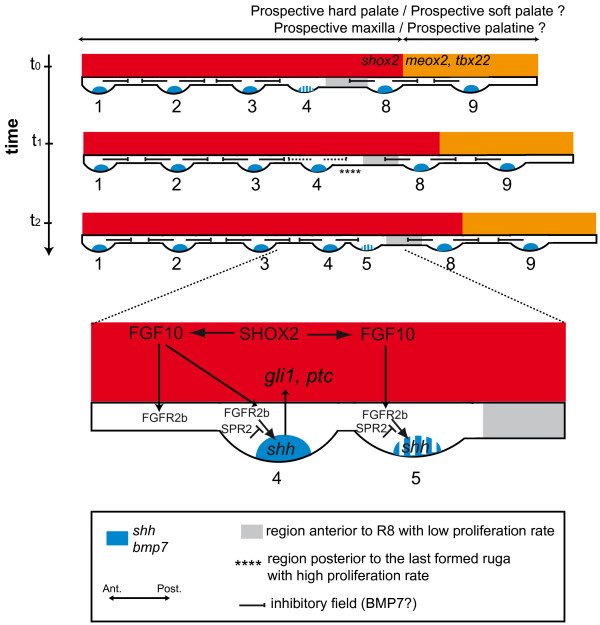
**A tentative model for ruga interposition and palate organization**. This model synthesizes data as shown in this paper together with data from the literature (see text for details).

Two other pathways have been directly involved in correct spacing and early formation of both hair and feathers: the EDA/EDAR pathway favors placode formation [35,36] while proteins of the BMP superfamily are responsible for establishing an inhibitory field around forming placodes [30,37]. *Eda *(encoding the EDA ligand) is expressed in the epithelium of the palate (SP, data not shown and [38]), and we previously have shown increased variability and occurrence of anomalies in the ruga pattern of Eda^Tabby ^(Eda^Ta^) null mutant mice [3]. However, we found no detectable expression of *Edar *in the developing palate, nor of IkB (SP, data not shown), which is a feedback transcriptional target of EDAR signaling in both hair and tooth [35,39,40]. These results thus do not favor a direct role of the EDA/EDAR pathway in rugae interposition. In contrast, we found that *Bmp7 *is expressed at the tip of the primitive rugae, in a pattern very similar to *Shh*. BMP7 is thus a good candidate for establishing an inhibitory field around rugae (Fig. 7).

FGF signaling is also involved in placode formation, although it has been formally demonstrated only for feathers but not yet for hair [41,42]. Here data from the literature suggest that it is actually another interesting pathway for ruga interposition. Indeed, epithelial FGFR2b is supposed to be activated by mesenchymal FGF10 and is necessary for *Shh *expression in rugae [10]. Moreover FGF10 beads can induce ectopic *Shh *expression, and *Fgf10 *knock-out mice lack rugae [10]. Finally, Welsh et al. [10,43] demonstrated that an inhibitor of FGF signaling, SPRY2, is necessary to restrict the proliferation rate in the palate and to achieve the normal pattern of rugae. FGF signaling might thus be one component of the activation-inhibition mechanism that we propose (Fig. 7). Alternatively, it could also play a more indirect role, by favoring palatal growth, which in turn can change the activation-inhibition equilibrium.

In conclusion, this preliminary survey of candidate pathways suggests that ruga interposition only involves a limited number of the pathways that have been implicated in hair and feather initiation. While the Wnt and EDA-EDAR pathways do not seem to be involved, the FGF and BMP pathways should be investigated in the future. But what are the reasons for these differences? At least three aspects shown in this article may be relevant. First, new rugae form from a region of already intermediate epithelium thickness (by comparison to the oral epithelium). Second, an obvious difference between rugae and hair or feather is the extent of the growth following the early steps of development. In this respect also, rugae more closely resemble scales. Here it is interesting to note that the growth difference between scales and feathers has been suggested to rely on different levels of activation of the Wnt/β-catenin pathway [9,31,32]. Third, new rugae do not form in a naïve field, like mouse primary hair (which arise periodically and simultaneously from a field with no apparent patterning information), nor in a field with an open side, like dorsal chicken feathers (for which a central row of feather buds is established and then rows are progressively added laterally), but they are interposed in a field between two already formed rugae. Similarly, it should be noted that formation of the very first rugae (i.e. formation of rugae 2 and 8, that was not addressed here) might require other pathways than ruga interposition.

### Rugae reveal the allometry of palatal growth and a new developmental boundary for palate development

We propose that ruga 8 in mouse and ruga 7 in the golden hamster, which are the rugae in front of which new rugae are added, can be called "boundary rugae". Indeed, the palatal shelf can be divided in two parts (Fig. 7): one, anterior to the boundary ruga, which A/P elongation seems rapid and where new rugae are added, and a second, posterior to the boundary ruga, which A/P elongation seems slower. We show that in both species, the boundary ruga marks the posterior end of *Shox2 *expression domain in the mesenchyme of the palatal shelf. In contrast, *Meox2 *and *Tbx22 *mesenchymal domains are found posteriorly to the boundary ruga. As a consequence of the different rate in A/P elongation between the anterior and the posterior part, this *Shox2/Meox2 *boundary seems to move posteriorly during development (Fig. 5), as it was initially noted by Li et al. (2007, [13]). In their proliferation assay, these authors found no obvious difference that could explain the different rate of expansion of the two domains. As a consequence, they proposed that mesenchymal *Meox2 *expressing cells could be recruited into the *Shox2 *domain to allow its expansion (these cells would then turn off *Meox2 *expression and turn on *Shox2 *expression). Importantly, such a recruitment model is less conceivable for explaining the different rate of expansion of the upper epithelial domains, where the boundary ruga seems to represent a stable morphological boundary. This leaves two possibilities: either only the epithelial boundary is stable, and would be able to reprogram the underlying mesenchyme while new cells are recruited, or there is a stable mesenchymal-epithelial boundary, and the *Shox2 *domain and *Meox2/Tbx22 *domains are separated compartments. Only lineage studies will help to discriminate between these two possibilities. In any case, our results clearly demonstrate that the boundary ruga marks a developmental boundary in the A/P organization of the palate. Of note, this organization may be shared with human since *Shox2 *expression is found in the anterior but not posterior palate of human embryos [14].

Finally, does this developmental boundary coincide with a known morphological boundary of the mouse head? In the adult head, the boundary ruga clearly does not coincide with the hard/soft palate limit, since both ruga 8 and 9 in the adult mouse (or ruga 7 and 8 in the hamster) are supported by the hard palate. However, we cannot exclude that, during the embryogenesis, the embryonic boundary ruga could actually correspond to the prospective hard/soft palate limit, but that this early relationship would be scrambled during later ontogenesis. In agreement with this view, *Shox2 *has been related to hard tissue morphogenesis (limb chondrogenesis [44]) while *Meox2 *has been related to soft tissue morphogenesis (limb muscle differentiation [45]). Alternatively, since in the adult mouse and hamster, the boundary ruga grossly sits on the top of the suture between the maxilla and the palatine bone, the identified developmental boundary might represent the prospective limit between the maxilla and the palatine. Again, lineage studies will be necessary to highlight the exact nature of this new developmental boundary.

### A working model for coordinated ruga interposition and A-P growth of the palate

We propose a working model, where ruga interposition is coordinated with A/P growth of the palate (Fig. 7). In this model, the palate is organized in two domains: an anterior domain, including the rugae between ruga 1 and the boundary ruga 8, where the mesenchyme is *Shox2 *positive, and a posterior domain, corresponding to the region posterior to ruga 8, and including both ruga 9 and the "soft palate", as generally defined, which is *Meox2 *and *Tbx22 *positive (but *Shox2 *negative). The anterior domain grows rapidly (for example under the influence of FGF signaling), which progressively increases the distance between already formed rugae. These rugae maintain an inhibitory field around them and, for example, the BMP7 protein, by diffusing from the tip of rugae, and this could help to maintain cells in a "non rugal" fate (Fig. 7, t_0_). We noticed that *Bmp7 *expression was turned on late during formation of one ruga (just as *Shh*). As a consequence, the inhibitory influence may be locally low just next to the last formed ruga, because A/P extension has moved more mature rugae away, and the last formed ruga still does not exert its inhibitory influence (see Fig. 7, t_0_). The cells there may then be able to adopt rugal fate and undergo proliferation to form the ruga thickening (Fig. 7, t_1_). Later on, *Shh *and *Bmp7 *become expressed at the tip of the newly formed ruga, and a true gap is formed. As previously mentioned, FGF signaling (from mesenchyme) probably participates in the induction and/or maintenance of *Shh *expression [10]. In turn, epithelial Shh signals to the underneath mesenchyme where *target genes like Gli1 *and *Ptc *are induced [23].

In summary, we propose that the continuous growth of the palate, coupled with a simple activation-inhibition mechanism, and a local disequilibrium (local low inhibition next to the last formed ruga), could generate oscillations that would be responsible for periodic and asymmetric ruga interposition. This working model could be tested in the future by combining mathematical modeling with functional test of candidate signaling molecules.

## Conclusion

In this article, we showed that rugae are sequentially added to the growing palate, in an interposition process that seems to be dependent on activation-inhibition mechanisms and reveals a new developmental boundary in the growing palate. These findings open two main directions. First, they define rugae as a new and simple model for studying not only origins of differences in development of skin appendages but also the patterning of serial structures. The latter aspect may benefit from already extensively studied systems such as somites and, in turn, could help to understand the patterning of molars, which are neighboring but much more complex serial structures also belonging to skin appendages. Of note, the complex question of molar segmentation was first addressed only very recently and could involve the two pathways pointed out for rugae in our discussion: the FGF and BMP pathways [46]. Second, these findings reveal that the secondary palate can be divided in two parts regarding the boundary ruga (ruga 8 in mouse), the growth of the anterior part being stronger. In the future, rugae will undoubtedly be helpful markers to further shed light on the A/P organization and allometric growth of the secondary palate.

## Methods

### Harvesting and staging of embryos

CD1 (ICR) adult mice were purchased from Charles River. Tabby mice were bred at the PBES (Lyon). The colony was established by inbreeding from a mating pair (B6CBACa Aw-J/A-Eda^Ta^/J-XO female and B6CBACa Aw-J/A male) obtained by the Jackson Laboratory (Bar Harbor, Maine).

Mouse females were mated overnight and the noon after morning detection of a vaginal plug was indicated as embryonic day (ED) 0.5. Pregnant mice were killed by cervical dislocation and embryos were harvested and weighted as described earlier [15]. Mouse heterozygous and homozygous Eda^Ta ^embryos (ED14.5–15.0) used in this study (Fig. 4) were obtained from Eda^Ta^/Eda^Ta ^females crossed with either Eda^Ta^/Y or +/Y males, and, when necessary, phenotypied based on the absence of primary hair follicle in homozygous *versus *heterozygous mutant embryos.

Pregnant hamster females (*Mesocricetus auratus*, HsdHan:AURA) were purchased from Harlan. They were killed by cervical dislocation following anesthesia and embryos were harvested and weighted as described for mouse embryos [15].

### Whole mount in situ hybridization

Heads of mouse embryos were fixed overnight in paraformaldehyde 4% at 4°C, dehydrated in a methanol series and kept for several weeks in 100% methanol. Heads were rehydrated and upper and lower jaw were separated before proceeding to whole mount *in situ *hybridization, which was done using standard methods [47]. Samples were imaged on a LUMAR stereomicroscope (Zeiss, PLATIM, Lyon). Following whole mount *in situ *hybridization, samples were embedded in paraffin and sectioned (5 μm). Sections were stained with nuclear fast red and imaged on a Zeiss microscope.

Several probes were as originally described: *Shh *[48]; *Shox2 *[49]; *Bmp7 *[50]. Other were made from RT-PCR products amplified from mouse or hamster embryonic total RNA and cloned in TOPO-PCRII (Invitrogen): *Meox2 *(5' GGTCCTGTGTTCCAACTCATC and 3' GAAGCGTTCCCTTTTTCACA); *Tbx22 *(5' ACAAAGTGGAAGCAGTGGCTCA and 3' GGCTGGATACCAATGGGAATGA), *ma-Shh *(5' CAAAAAGCTGACCCCTTTAGCCT and 3' AGGAAGGTGAGGAAGTCGCTGTA), *ma-Meox2 *(5' ATGGAACACCCSCTCTTTGG and 3' CCACACTTTCACCTGTCTTTCAGT).

### Assessment of epithelium thickness and proliferation rate along the A/P axis of the palatal shelve

In order to trace the rapid dynamic of rugae development at ED13.5, we used CD1 (ICR) mouse embryos exhibiting a gradient series of weights: 179 mg, 192 mg, 208 mg. Heads were fixed individually in Bouin-Holland fluid and routinely embedded in paraffin. Series of 5 μm thick frontal sections were prepared and stained with alcian blue-hematoxylin-eosin.

For every two sections, we took into consideration the epithelium covering the oral surface of embryonic maxilla in a region of fixed area representing the central part of a palatal shelf. This fixed area was centered on a point situated at mid distance between the palatal shelf margin and either the lip furrow (in lip region) or the centre of a base of a tooth germ (in a cheek region) using Leica DMLB microscope.

In this fixed area of epithelium, we measured 3 parameters on each second section: (1) Thickness of the epithelium was measured using an objective 100× and a drawing chamber. After the projection of a section on a white surface (resultant magnification 800×), the epithelium thickness was measured by a straight edge. If the epithelium exhibited thickness and morphology of a common epithelium lining the oral cavity (i.e. a single layer of basal cells and a single layer of flat superficial cells, total thickness less than 18 μm), then the section was classified as part of a gap between rugae (white in Fig. 3). Otherwise, the section was considered as part of a ruga (dark grey in Fig. 3). The very special region anterior to ruga 8, where new rugae form, was treated separately (light grey in Fig. 3); (2) A number of epithelial cells in mitosis was counted on pictures captured using an objective 100×/1.25 and a Leica DC480 camera. The cells in mitosis and the mitosis phase were then verified directly in the microscope. Only the cells from early metaphase to early telophase were considered. (3) A total number of epithelial cells. All present epithelial cells were counted on identical images that were used for the counting of mitotic cells (see above). Finally, for gaps, rugae and the region of new ruga formation (determined on the basis of epithelium thickness – see above), we calculated a "mitotic index" as the percentage of the total number of mitotic cells in the total number of cells for the concerned sections.

### Ki67 immunostaining on cryosections

The heads were dissected from CD1 (ICR) mouse embryos at ED 13.5 (weight range 204–210 mg) and frozen immediately in O.C.T. Tissue Tek (Sakura) diluted 1:1 with Hank's balanced solution. Frozen sections were postfixed in 4% PFA for 10 minutes and processed by a Citrate buffer epitope retrieval method: 10 minutes boiling in 10 mM sodium citrate buffer pH 6, 0.05% Tween 20. The sections were then incubated at RT overnight with primary antibody Ki67 (Rabbit polyclonal IgG to Ki67, Abcam, #Ab15580) diluted 1:1000 in 1% BSA. The secondary antibody (Alexa Fluor^® ^568 goat anti-rabbit IgG, Invitrogen, #A11036) was diluted 1:800 in 1% BSA and incubated at RT for 45 min. Nuclei were stained with Prolong^® ^Gold antifade reagent with DAPI (Invitrogen). The images were made using a microscope Leica DMI 6000B and camera DFC 490.

### Ki67 whole mount immunostaining

Palatal halves were dissected in PBS from CD1 (ICR) embryos at 13.5 ED and fixed overnight in 4% PFA in PBS. Whole mount immunostaining was performed as described in Nagy et al., 2003. Anti-Ki67 (clone SP6) (1:200 dilution) was obtained from Lab Vision (#RM-9106) and detected with anti-rabbit-Alexa88 (Molecular Probes). Following, nuclei were labeled with Draq5^TM ^as recommended by the manufacturer (Biostatus). Palatal shelves were imaged on a confocal microscope (Zeiss LSM510, PLATIM, Lyon).

### Measurements in wild type adult palates

The sample, composed of 82 adult wild type mice of B6CBACa genetic background, was already described in our previous paper [3]. Palates were imaged using a Leica MZ 16 stereomicroscope (Leica, Wetzlar, Germany) and a LEICA DFC 320 CCD-camera. As left and right rugae originate independently, palatal halves were treated separately (hence n = 164). The distance between rugae 7 and 8 was the mean value of three measures taken at three different positions between the basements of the two rugae (1-lateral extremity of ruga 7 straight to ruga 8; 2-center of ruga 7 straight to ruga 8; 3-medial extremity of ruga 7 straight to ruga 8), using the Optimas software.

## Abbreviations

ED: Embryonic Day; A/P: Anterior/Posterior; WISH: whole mount *in situ *hybridization

## Authors' contributions

SP carried out *Shh *whole mount *in situ *hybridization on wild type and tabby mice and Ki67 whole mount immunolabeling, participated to the work on A/P regionalization, and generally conceived of the study, designed and coordinated it, and drafted the manuscript. JP characterized *Shh *expression on sections and worked setting up tissue culture experiments that were not conclusive enough to be included. AM did the work with hamster embryos, with TOP-GAL mice and the initial work on A/P regionalization. MR carried out mitotic countings and Ki67 labeling on sections. AL carried out the whole mount *in situ *hybridization and sections concerning the A/P regionalization of the palate. LB carried out *Shh *whole mount *in situ *hybridization on Eda^Ta ^mice. CC and LV carried out the analysis of ruga 7 – ruga 8 distance in adults. RP and VL both participated to the study conception and design, and helped to revise the manuscript.

All authors read and approved the final manuscript.
